# Antimicrobial peptide LL-37 increases rhinovirus-induced interferon β expression in human airway epithelial cells through a Ca^2+^-dependent mechanism

**DOI:** 10.1016/j.bbrep.2025.102105

**Published:** 2025-06-21

**Authors:** Samuel Cerps, Sangeetha Ramu, Olof Gidlöf, Mandy Menzel, Karl Swärd, Lena Uller, Bengt-Olof Nilsson

**Affiliations:** a Department of Experimental Medical Science, Lund University, BMC D12, SE-22184, Lund, Sweden; b Department of Cardiology, Clinical Sciences, Lund University, Skåne University Hospital, BMC D12, SE-22184, Lund, Sweden

**Keywords:** Cathelicidin, Endosome, Host defense peptide, Innate immunity, Interferons, Rhinovirus

## Abstract

The human cathelicidin LL-37 is active against both bacteria and viruses, but it also shows immunomodulatory properties. Here, we assess the impact of LL-37 on viral signaling in human airway epithelial BEAS-2B cells infected with the respiratory pathogen rhinovirus (RV). We show that LL-37 (4 μM) enhances RV-induced expression of interferon β (IFNβ) transcript and reduces viral-load. LL-37-evoked potentiation of RV-stimulated IFNβ does not involve up-regulation of the classical viral TLR3, MDA5 and RIG-I receptors. Moreover, the LL-37-induced stimulation of IFNβ expression in the presence of RV is abolished by chloroquine, an inhibitor of endosomal acidification. Interestingly, RV + LL-37-induced stimulation of IFNβ is observed in the absence but not in the presence of the Ca^2+^ chelating agent EGTA, indicating that Ca^2+^ is critical for this effect. Indeed, we demonstrate that LL-37 increases intracellular [Ca^2+^] in cells loaded with the fluorescent Ca^2+^ indicator Fluo-4 AM. Furthermore, we reveal that treatment with RV in combination with the Ca^2+^ ionophore A23187 promotes IFNβ expression, showing the importance of Ca^2+^. In conclusion, we demonstrate that LL-37 acts in synergy with RV to enhance IFNβ expression and that this effect involves LL-37-induced increase in intracellular [Ca^2+^].

## Highlights

• LL-37 reduces rhinovirus-load in human airway epithelial cells.
• LL-37 potentiates rhinovirus-induced IFNβ mRNA expression.
• Inhibition of endosomal acidification abolishes LL-37-induced potentiation of IFNβ
• LL-37 elevates intracellular [Ca^2+^].
• LL-37-induced potentiation of IFNβ is observed in presence but not absence of Ca^2+^.

## 1 Introduction

The pro-form of LL-37, hCAP18 (encoded by the *CAMP* gene), is produced by human white blood cells and epithelial cells and processed extracellularly to the antimicrobial peptide LL-37 [[1], [2], [3]]. LL-37 permeabilizes the bacterial cell wall and thereby shows direct activity against both gram-positive and gram-negative bacteria [[4], [5], [6]]. Furthermore, LL-37 binds and inactivates bacterial endotoxins, demonstrating that the peptide is active against bacteria through multiple mechanisms [[7], [8], [9], [10]]. Importantly, LL-37 not only possesses antimicrobial effects, but it also modulates the immune system via both pro- and anti-inflammatory mechanisms [11,12].

Beyond its antibacterial activity, LL-37 targets different types of viruses [[13], [14], [15], [16], [17]]. The peptide shows antiviral activity against respiratory pathogens such as influenza A virus, respiratory syncytial virus, and rhinovirus (RV), suggesting that LL-37 is of pathophysiological importance in the lungs and upper airways [[18], [19], [20]]. LL-37 is widely recognized to possess antiviral activity against many types of enveloped viruses, but the peptide also shows antiviral activity towards non-enveloped viruses such as RV, which is a virus strongly associated with respiratory tract infections and asthma exacerbations [[20], [21], [22]]. Interferon β (IFNβ) belongs to the type I interferons and is produced by host cells in response to viral components and activation of intracellular viral receptors. Type I interferons activate the immune system, and they reduce protein synthesis and trigger apoptosis in infected host cells to reduce virus replication and spread [23]. Importantly, the underlying mechanism of action behind LL-37-induced antiviral activity towards RV is not fully understood, and it is not known whether LL-37 has an impact on the RV-induced interferon response.

Recently, LL-37 has been reported to potentiate synthetic double-stranded (ds) RNA (poly I:C)-induced pro-inflammatory cytokine production through a mechanism that seems to involve enhanced expression of the viral receptor TLR3, but it is not known whether LL-37 modulates viral receptor expression and the inflammation response also in the presence of intact virus [24]. In skin keratinocytes, treatment with LL-37 alone has no effect on IFNβ expression, but it strongly enhances poly I:C-induced IFNβ production, suggesting that LL-37 potentiates the effect of poly I:C on IFNβ expression in this cell type [25]. In viral airway infection, white blood cells rich in hCAP18/LL-37 are recruited to the site of infection [11]. Thus, it is of great importance to assess the interplay between RV and LL-37, and their effects on the airway epithelial immune response.

Here, we assess the impact of LL-37 on RV-induced IFNβ expression and investigate its mechanisms of action in human airway epithelial BEAS-2B cells. We demonstrate that LL-37 potentiates RV-induced IFNβ mRNA expression through a mechanism that involves enhanced intracellular [Ca^2+^], and we also show that LL-37 reduces the RV-load.

## 2 Materials and methods

### 2.1 Cells and cell culture

The human bronchial epithelial cell line BEAS-2B was purchased from ATCC. Cells were cultured in RPMI-1640 medium (Thermo Fisher Scientific) supplemented with fetal bovine serum (10 %, Thermo Fisher Scientific) and antibiotics (penicillin 50 U/ml and streptomycin 50 μg/ml, both from Thermo Fisher Scientific) in a water-jacketed incubator at 37 °C under 5 % CO_2_ in air. For qPCR analysis, 40000 cells were seeded in each well of 12-well plates, and for ELISA and dot blot analysis, 140000 cells were seeded in each well of 6-well plates 4 days prior to experiments. For intracellular Ca^2+^ measurements, 10000 cells were seeded in each well of 96-well plates one day prior to experiments. Cells were used for experiments at confluence assessed by phase contrast light microscopy (Nikon TMS, Nikon). In some experiments, cells were cultured in RPMI-1640 medium ([Ca^2+^] = 0.42 mM) supplemented with EGTA (0.5 mM) to bind free Ca^2+^ ions and thus establish Ca^2+^-free culture conditions.

### 2.2 Viral infection

In preliminary experiments, cells were incubated with increasing concentrations from 0.1 MOI up to 0.25 MOI of RV1B for 24 h to establish the highest dose of virus particles where no or small negative effects on cell morphology were observed. Rhinovirus RV1B (Human rhinovirus 1B strain B632, catalog number VR-1645, ATCC) was grown in Ohio HeLa cells (European Collection of Cell Cultures) and obtained from clarified cell lysates as previously described (1.58 × 10^7^ tissue culture infectious dose (TCID50)/mL) [26]. Cell morphology was assessed in a Nikon TMS phase contrast light microscope. Based on this analysis, we chose 0.15 MOI. For viral infection, cells were incubated with 0.15 MOI of RV1B for 1 h in room temperature during shaking. Infected cells were then washed in PBS and treated with or without LL-37 (4 μM) for 24 and 48 h. We used 4 μM LL-37 in all our experiments, since this concentration is below what has been reported previously (∼10 μM) to be cytotoxic in airway epithelial cells [27]. Neither RV1B nor LL-37 (4 μM) had an impact on cellular morphology.

### 2.3 Quantitative real-time RT-PCR

Total RNA was isolated from cell lysates using RNeasy Plus mini kit (Qiagen) according to the manufacturer’s protocol. Total RNA levels and RNA quality were analyzed spectrophotometrically (NanoDrop 2000c, Thermo Fisher Scientific) and 1 μg RNA was reversely transcribed into cDNA with High-Capacity cDNA Reverse Transcription Kit (Applied Biosystems, Thermo Fisher Scientific). Amplification was performed using the AriaMx real-time PCR system (Agilent Technologies). TaqMan primers (Thermo Fisher Scientific) for the following transcripts were used: UBC, IFNB1, TLR3, MDA5, RIG1, and RV1B. The −ΔΔCt method was applied for quantification using UBC (ubiquitin C) as housekeeping gene.

### 2.4 ELISA analysis, dot blot analysis and total protein measurement

For ELISA analysis of IFNβ levels in cell supernatants, culture medium was removed and cells washed in ice-cold PBS. Cells were detached from the plate in ice-cold PBS by cell scrapers (Sarstedt). Cell supernatants were prepared by sonication (2 × 10 s) on ice and centrifugation (5000 *g*, 5 min, 4 °C). The samples were stored at −80 °C until analysis. IFNβ protein levels were determined by ELISA according to instructions by the manufacturer (R&D Systems, catalog number DY814) and normalized to the total protein concentration in each sample.

For dot blot analysis, 1 μl of the cell supernatant was spotted on nitrocellulose membranes (0.2 μm pore size, Bio-Rad). PBS was included as blank. Membranes were blocked in 1 % casein buffer (Bio-Rad) and incubated with a polyclonal rabbit IFNβ antibody from Novus Biologicals (catalog no. NBP-77288) diluted 1:350 for 48 h. After incubation with the primary antibody, membranes were washed 3 times in Tris-buffered saline containing 0.1 % Tween 20 (Bio-Rad) and incubated with horseradish peroxidase-conjugated anti-rabbit IgG (Cell Signaling, catalog no. 7074) diluted 1:5000 for 2 h. Membranes were washed 3 times in Tris-buffered saline (0.1 % Tween 20) and incubated with West Femto chemiluminescence reagent (Thermo Fisher Scientific). IFNβ immunoreactivity was visualized with a LI-COR Odyssey instrument (LI-COR Biosciences).

Total protein concentration in the cell supernatants was determined with a total protein assay (DC protein assay, Bio-Rad). Each sample was analyzed in duplicate.

### 2.5 Measurement of intracellular Ca^2+^ concentration

Cells were seeded in 96-well plates with black bottom (Corning) and allowed to attach to the surface of the well and equilibrate overnight. Cells were loaded with the Ca^2+^-indicator Fluo-4 AM (5 μM, Invitrogen) in HEPES (N-2-hydroxyethylpiperazine-N′-2-ethanesulfonic acid)-buffered salt solution with Pluronic F-127 (0.05 %, Sigma-Aldrich) for 60 min and then carefully washed two times in HEPES solution. Cells were treated with LL-37 (4 μM) or vehicle in HEPES solution with a physiological concentration of Ca^2+^ (2.5 mM), and cellular fluorescence determined in a CLARIOstar Plus multi-mode microplate reader (BMG Labtech).

### 2.6 Agents

LL-37 and A23187 were purchased from Bachem and Sigma-Aldrich, respectively. A23187 is a carboxylic acid acting as a carrier for Ca2+ over biological membranes. Both LL-37 and A23187 were dissolved in DMSO. Chloroquine was from Sigma-Aldrich and dissolved in PBS. Controls received vehicle (DMSO and PBS, 0.1 %) as appropriate.

### 2.7 Statistics

Data were analyzed using GraphPad Prism9 (GraphPad Software) and summarized data are presented as means ± standard error of the mean (SEM). Statistical significance was computed using Student’s two-tailed *t*-test for single comparisons between two groups and one-way ANOVA followed by Holm-Sidak’s post-hoc analysis for multiple comparisons as appropriate.

## 3 Results

### 3.1 LL-37 potentiates RV-induced IFNβ transcript expression in BEAS-2B cells

**Fig. 1:**
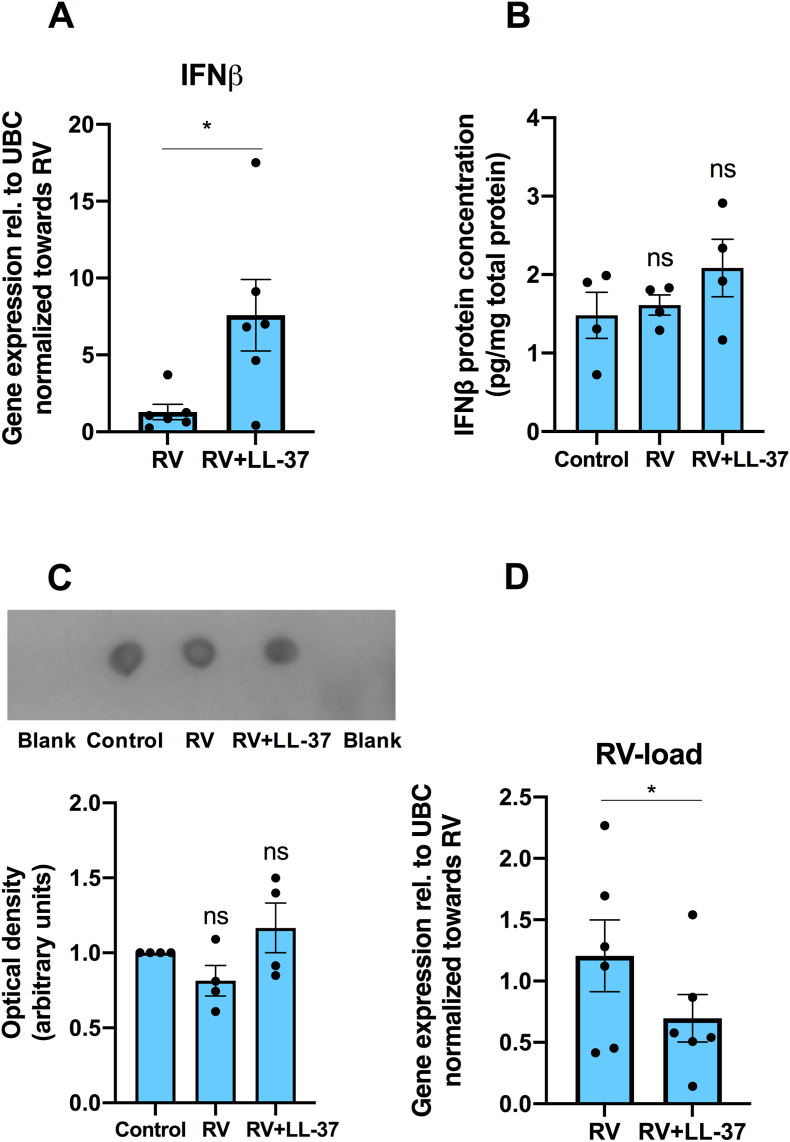
**LL-37 potentiates RV-induced IFNβ mRNA expression and attenuates RV-load in BEAS-2B cells. (A**–**D)** Cells were pre-incubated with LL-37 (4 μM) for 1 h before RV (0.15 MOI) was included. After 1 h with RV, cells were incubated for 24 (**A, D**) and 48 h (**B, C**) in the continuous presence or absence of LL-37. Expression of IFNβ and RV transcripts were determined by qPCR (**A, D**). (**A**) Effects of LL-37 on RV-induced IFNβ mRNA expression. (**B**) IFNβ protein concentration in cell supernatants was determined with ELISA and normalized to total protein contents in each sample. (**C**) IFNβ immunoreactivity in cell supernatants was analyzed with dot blot applying PBS as blank. (**D**) Effects of LL-37 on RV-load. DMSO (0.1 %) was included as vehicle control. Values are presented as means ± SEM. ∗ represents P < 0.05. ns = not significant vs. vehicle control. Statistical significance was determined with Student’s two-tailed paired *t*-test for single comparisons between two groups and one-way ANOVA followed by Holm-Sidak’s post-hoc analysis for multiple comparisons as appropriate.

In the first experiments, we assessed the effects of infection with RV alone (0.15 MOI) and RV in combination with LL-37 (4 μM) on both IFNβ mRNA expression and IFNβ protein production in BEAS-2B cells. In unstimulated control cells, no IFNβ transcript was detected, whereas IFNβ transcript was detectable in cells infected with RV for 24 h. IFNβ mRNA was undetectable also in cells stimulated with LL-37 (4 μM) alone. Infection with RV in combination with LL-37 (4 μM) for 24 h increased IFNβ transcript levels by about 6-fold (P < 0.05) compared to RV alone (Fig. 1A). Next, we assessed the effects of infection with RV and RV + LL-37 on IFNβ protein levels in BEAS-2B cell supernatants. ELISA analysis showed that infection with RV and RV + LL-37 (4 μM) for 48 h had no effects on IFNβ protein levels (Fig. 1B). For the same experiments and samples, we also performed dot blot analysis applying another IFNβ antibody. Dot blot analysis demonstrated that infection with RV and the combination of RV and LL-37 (4 μM) had no effects on IFNβ protein levels confirming the ELISA data (Fig. 1C). Hence, our results show, using 2 different techniques and antibodies, that RV-induced IFNβ-transcript expression and its potentiation by LL-37 (4 μM) does not result in elevated cellular IFNβ protein levels at 48 h, although mRNA levels for IFNβ are increased several folds at 24 h.

**Supplemental Fig. 1:**
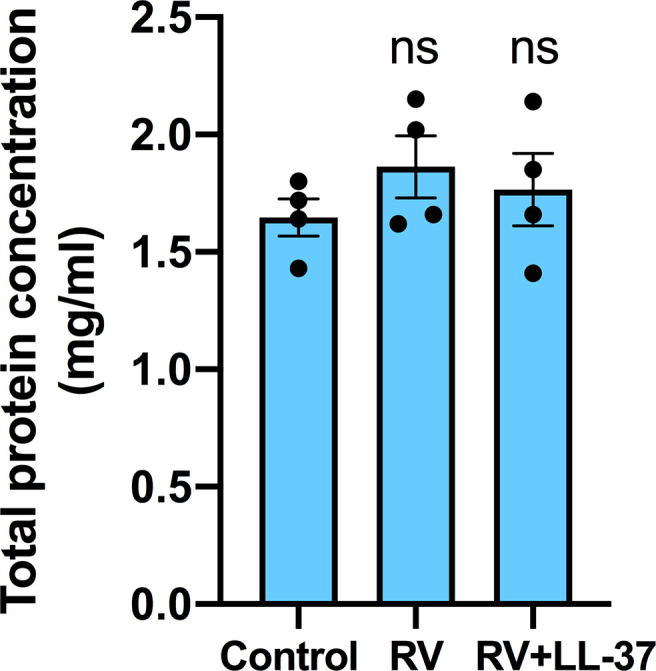
**RV and RV** + **LL37 have no effects on total protein levels.** Total protein concentration in BEAS-2B cell lysates was determined at 48 h using a total protein assay. ns = not significant vs. vehicle control. Please, see legend to Fig. 1 for further information about experimental protocol and statistics.

Importantly, infection with either RV alone or the combination of RV and LL-37 (4 μM) had no effect on total protein concentrations in the cell supernatants, showing that infection with RV and RV + LL-37 do not affect cell proliferation and cell turnover (Supplemental Fig. 1). Interestingly, stimulation with LL-37 (4 μM) for 24 h reduced the RV-load by about 40 % (P < 0.05), suggesting a direct LL-37-induced antiviral effect, although LL-37 also may reduce RV-load indirectly via induction of IFNβ expression (Fig. 1D).

### 3.2 LL-37 has no effect on RV-induced TLR3, MDA5 and RIG-I mRNA expression

**Fig. 2:**
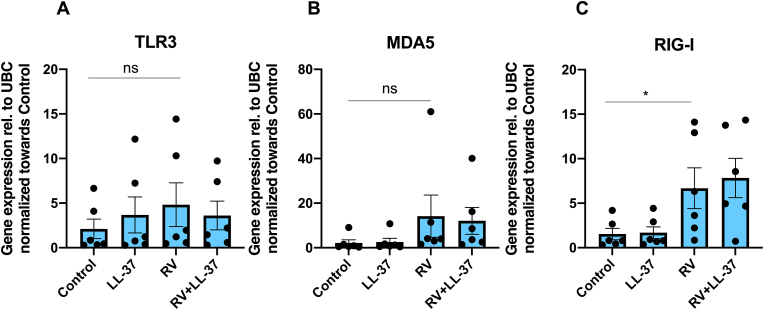
**LL-37 has no effect on RV-induced viral receptor expression in BEAS-2B cells. (A**–**C)** Cells were pre-incubated with LL-37 (4 μM) for 1 h before RV (0.15 MOI) was included. After 1 h with RV, cells were incubated for 24 h in the continuous presence or absence of LL-37. (**A**) TLR3, (**B**) MDA5 and (**C**) RIG-I transcript expressions were determined by qPCR. DMSO (0.1 %) was included as vehicle control. Values are presented as means ± SEM. ∗ represents P < 0.05 vs. vehicle control. ns = not significant vs. vehicle control. Statistical significance was determined with one-way ANOVA followed by Holm-Sidak’s post-hoc analysis for multiple comparisons as appropriate.

Next, we assessed if RV + LL-37-induced up-regulation of the IFNβ response is associated with enhanced viral receptor expression in BEAS-2B cells. Infection with RV alone (0.15 MOI) for 24 h enhanced mRNA expression for the intracellular virus receptor RIG-I by about 7-fold (P < 0.05), with no effect on either TLR3 or MDA5 transcript (Fig. 2A–C). Treatment with LL-37 (4 μM) in combination with RV had similar effects as infection with RV alone for all three receptors (Fig. 2A–C). Treatment with LL-37 (4 μM) alone had no effect on TLR3, MDA5 and RIG-I expression (Fig. 2A–C).

### 3.3 LL-37 increases intracellular Ca^2+^ concentration in BEAS-2B cells

**Fig. 3:**
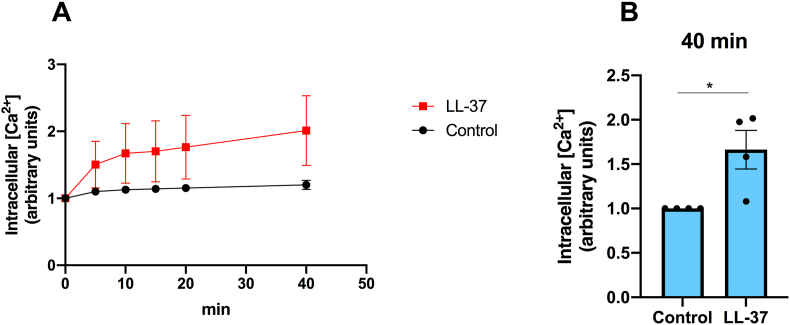
**Stimulation with LL-37 increases intracellular Ca^2+^ concentration in BEAS-2B cells. (A, B)** Cells were loaded with the fluorescent Ca^2+^ indicator dye Fluo-4 AM and stimulated with or without LL-37 (4 μM) in Ca^2+^-containing (2.5 mM) HEPES solution. (**A**) Fluorescence was assessed continuously every 5 min for 40 min in a fluorescence microplate reader, and the time curve plotted and normalized to background fluorescence before LL-37 or vehicle was introduced. (**B**) Fluorescence determined at the endpoint (40 min) and compared to vehicle control. The fluorescence signal was normalized to fluorescence in control cells for each of the 4 independent experiments. DMSO (0.1 %) was included as vehicle control. Values are presented as means ± SEM. ∗ represents P < 0.05 vs. vehicle control determined with Student’s two-tailed unpaired *t*-test for single comparisons between two groups as appropriate.

**Supplemental Fig. 2:**
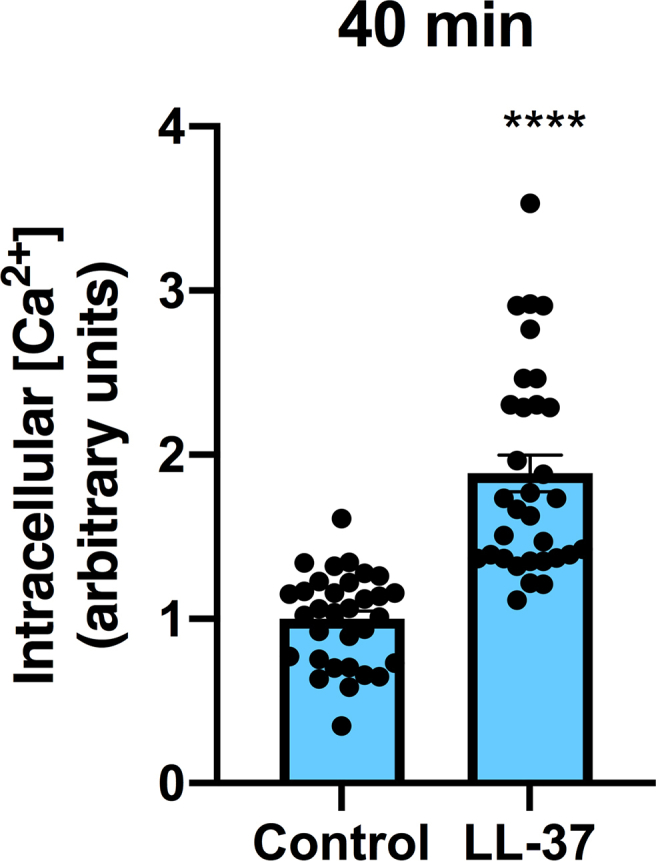
**Stimulation with LL-37 for 40 min increases intracellular Ca^2+^ concentration.** The complete data set of fluorescence in 4 independent experiments with 8 replicates (culture wells) for each experiment (n = 32 in each group). ∗∗∗∗ represents P < 0.0001. Please, see legend to Fig. 3 for further information about experimental protocol and statistics.

LL-37 (4 μM) has been shown to enhance intracellular [Ca^2+^] in human osteoblast-like MG63 cells [28]. In the next experiments, we therefore investigated if LL-37 increases intracellular [Ca^2+^] also in BEAS-2B cells. Cells were cultured in 96-well plates, loaded with the fluorescent Ca^2+^ indicator dye Fluo-4 AM, and fluorescence was determined in a microplate reader. The intracellular [Ca^2+^] increased with time in response to 4 μM LL-37 (Fig. 3A). In control cells not treated with LL-37, the intracellular [Ca^2+^] increased slightly with time, but to a smaller extent compared to LL-37-stimulated cells (Fig. 3A). At the end of the experiment (40 min), the intracellular [Ca^2+^] was about 70 % higher (P < 0.05) in the presence than in the absence of 4 μM LL-37 (Fig. 3B). In addition to the normalized data from the 4 independent experiments presented in Fig. 3B, the complete data set of Fluo-4 AM/Ca^2+^ fluorescence in control cells and cells stimulated with LL-37 (4 μM) at 40 min in the 4 independent experiments with 8 replicates in each group and each experiment (n = 32 culture wells in each group) clearly demonstrates that LL-37 increases intracellular [Ca^2+^] (Supplemental Fig. 2).

### 3.4 LL-37 potentiates RV-induced IFNβ expression in the absence but not in the presence of EGTA

**Fig. 4:**
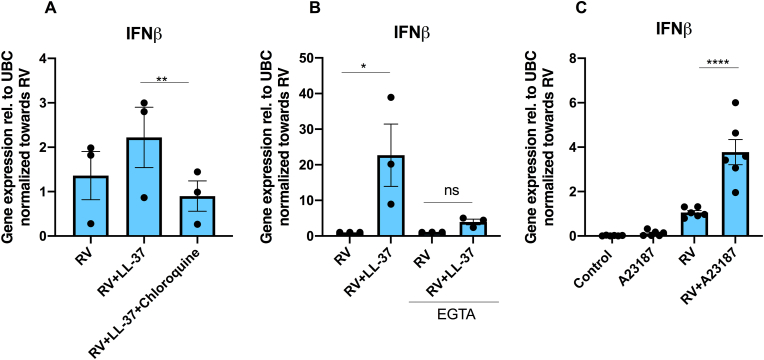
**LL-37 potentiates RV-induced IFNβ expression in the absence but not in the presence of EGTA. (A)** Effects of the endosome acidification inhibitor chloroquine on LL-37-induced IFNβ transcript expression. BEAS-2B cells were pre-incubated with LL-37 (4 μM) + chloroquine (2 μg/ml) for 1 h before RV (0.15 MOI) was included. After 1 h with RV, cells were incubated for 24 h in the continuous presence or absence of LL-37 + chloroquine. (**B**) Effects of LL-37 on IFNβ mRNA expression in the absence or presence of EGTA. BEAS-2B cells were pre-incubated with LL-37 (4 μM) in the absence or presence of 0.5 mM EGTA for 1 h before RV (0.15 MOI) was included. After 1 h with RV, cells were incubated for 24 h in the continuous presence or absence of LL-37 and EGTA. (**C**) Stimulation with the Ca^2+^ ionophore A23187 (10 μM) in combination with RV for 24 h potentiates RV-stimulated IFNβ transcript expression in BEAS-2B cells. A23187 was introduced 1 h prior to RV. After 1 h with RV (0.15 MOI), cells were incubated for 24 h in the continuous presence or absence of A23187. DMSO and PBS (0.1 % for both) were included as vehicle controls. Values are presented as means ± SEM. ∗, ∗∗ and ∗∗∗∗ represent P < 0.05, P < 0.01 and P < 0.0001 determined with one-way ANOVA followed by Holm-Sidak’s post-hoc analysis for multiple comparisons as appropriate. ns = not significant.

The potentiation by LL-37 (4 μM) of the RV-mediated IFNβ mRNA response was eliminated by the endosomal acidification inhibitor chloroquine (2 μg/ml) (Fig. 4A). Interestingly, chloroquine has been reported to inhibit Ca^2+^ channels and reduce intracellular [Ca^2+^] in B lymphocytes, suggesting that it may antagonize the LL-37-induced potentiation of IFNβ expression via this mechanism [29].

To assess if LL-37-induced increase in intracellular [Ca^2+^] may be associated with RV-stimulated IFNβ expression, we infected BEAS-2B cells with RV alone and RV + LL-37 (4 μM) in the presence or absence of the Ca^2+^ chelator agent EGTA. In order to bind all Ca^2+^ ions in the cell culture media, EGTA was added to the medium to 0.5 mM, which exceeds the concentration of Ca^2+^ in the RPMI-1640 cell culture medium (0.42 mM). Therefore, this concentration of EGTA effectively chelates the majority of extracellular Ca^2+^ ions. The combined treatment with RV (0.15 MOI) and LL-37 (4 μM) increased cellular IFNβ mRNA levels by about 20-fold (P < 0.05) compared to RV alone in culture medium with no EGTA, whereas LL-37 (4 μM) had no potentiating effect on the RV response in the medium containing EGTA (Fig. 4B). Infection with RV (0.15 MOI) alone for 24 h in the presence of EGTA did not affect IFNβ transcript expression compared to the RV response in the absence of EGTA (Fig. 4B). Next, we assessed if raising cytosolic [Ca^2+^] with the well-established Ca^2+^ ionophore A23187 mimicked the effect of LL-37. Infection with RV in combination with A23187 (10 μM) for 24 h increased IFNβ transcript levels about 4-fold (P < 0.0001) compared to RV alone (Fig. 4C). Hence, both LL-37 and A23187 potentiates RV-induced IFNβ expression severalfold. Treatment with A23187 alone had no effect on IFNβ expression (Fig. 4C).

## 4 Discussion

Here, we show for the first time that LL-37 potentiates RV-induced IFNβ expression in human airway epithelial cells, suggesting that LL-37 may protect against RV-induced airway infections through this mechanism. Interestingly, LL-37 reduces the viral load by about 40 %, arguing that LL-37 affects RV replication. In pulmonary infection, white blood cells, such as neutrophils, are recruited to the site of infection and these cells release large amounts of LL-37. LL-37 is detected in bronchoalveolar lavage (BAL) fluid of newborn infants, and interestingly LL-37 levels are increased in BAL fluid collected from children with airway infection, suggesting that our present findings are of *in vivo* relevance [30].

We demonstrate, by using both omission of extracellular Ca^2+^ with EGTA and mobilization of cytosolic Ca^2+^ with A23187, that LL-37-induced potentiation of RV-stimulated IFNβ expression involves Ca^2+^. Importantly, we also show that LL-37 increases intracellular [Ca^2+^], arguing that LL-37 indeed acts through raising cytosolic [Ca^2+^]. Notably, LL-37 has been shown to induce LDH release, and hence it permeabilizes the plasma membrane, an observation made in different types of human host cells [31]. Hence, we believe that LL-37 permeabilizes BEAS-2B cells and forms pores in their plasma membrane, allowing Ca^2+^ to flow into the cell along its concentration gradient. Interestingly, previous investigations demonstrate that increases in intracellular Ca^2+^ oscillations are coupled to enhanced expression of IFNβ in macrophages [32], and here we show that LL-37-induced potentiation of RV-mediated IFNβ expression is dependent on Ca^2+^, providing further evidence that Ca^2+^ is important for the IFNβ response. The transcription factor interferon regulatory factor 3 (IRF3) is important for transcription of the IFNβ gene, and IRF3 is activated by phosphorylation in a process dependent on Ca^2+^, suggesting that RV + LL-37 may promote IFNβ expression via this mechanism [33].

We could not detect an LL-37-induced potentiation of RV-stimulated IFNβ protein production, although IFNβ transcript was elevated by the combined treatment with LL-37 and RV, representing a limitation of the study. One possibility is that the RV + LL-37-induced increase in mRNA levels for IFNβ compared to RV alone is not high enough to affect the protein levels. Another option is that the time window for the detection of an increase in protein is narrow, and that we therefore miss it. Discrepancies between transcript and protein expression have been attributed to buffering of mRNA fluctuations masking changes in protein levels, a phenomenon which may occur in our experimental system [34]. In the present study, we examine IFNβ transcript expression at one time point (24 h) and IFNβ protein levels at another (48 h), apply a single concentration of LL-37, and use one cell line, representing limitations of the study.

In the present study, we used LL-37 at a concentration of 4 μM throughout. This concentration of LL-37 is in the same range as the local concentration LL-37 at the site of infection/inflammation in patients suffering from inflammatory conditions such as psoriasis and periodontitis [35,36], and in the same range as determined in sputum of COPD patients with colonization of pathogenic bacteria [37]. Hence, we suggest that 4 μM LL-37 is of *in vivo* importance also in infected/inflamed airways. Treatment with 4 μM LL-37 exerts low cytotoxicity in BEAS-2B cells, and thus it is unlikely that LL-37-induced stimulation of IFNβ production involves LL-37-induced cytotoxicity [38].

Intriguingly, we observe no LL-37-induced potentiation of RV-stimulated TLR3, MDA5 and RIG-I expression, arguing that LL-37 does not enhance RV-induced IFNβ expression via upregulation of virus receptors, although it should be kept in mind that LL-37 shows antiviral effects which may have an impact on the interpretation of these results. Previously, it has been demonstrated that stimulation with LL-37 in combination with poly I:C upregulates TLR3 expression in vascular smooth muscle cells [24]. Notably, poly I:C is an agonist on TLR3, whereas RV acts in a much wider and more unspecific manner involving a concerted action of many pattern recognition receptors and down-stream signaling [39], suggesting that stimulation with RV may trigger signaling pathways which antagonize dsRNA-induced up-regulation of TLR3 in a redundant manner.

In summary, we disclose that LL-37 acts in synergy with RV to promote airway epithelial cell IFNβ expression, and that this effect involves Ca^2+^ and elevated intracellular [Ca^2+^]. Furthermore, we demonstrate that LL-37 reduces the RV-load, and thus we propose that LL-37 may have a beneficial effect in viral airway infections through both these mechanisms.

## CRediT authorship contribution statement

**Samuel Cerps:** Writing – review & editing, Visualization, Methodology, Investigation, Formal analysis, Conceptualization. **Sangeetha Ramu:** Writing – review & editing, Methodology, Investigation. **Olof Gidlöf:** Writing – review & editing, Methodology, Investigation. **Mandy Menzel:** Writing – review & editing, Methodology, Conceptualization. **Karl Swärd:** Writing – review & editing, Methodology, Investigation, Funding acquisition. **Lena Uller:** Writing – review & editing, Supervision, Resources, Methodology, Funding acquisition, Conceptualization. **Bengt-Olof Nilsson:** Writing – review & editing, Writing – original draft, Supervision, Resources, Methodology, Investigation, Funding acquisition, Formal analysis, Conceptualization.

## Funding

This study was supported by grants from the Alfred Österlund Foundation to BON, the Alfred Österlund Foundation to LU, the 10.13039/100016409Gyllenstiernska Krapperup Foundation (grant number KR2024-0001) to BON, the Heart-Lung Foundation (grant number 20210440 to LU and grant number 20230255 to KS), and the 10.13039/501100004359Swedish Research Council (grant numbers 2023-02030 and 2020-00922) to LU.

## Declaration of competing interest

The authors declare that they have no known competing financial interests or personal relationships that could have appeared to influence the work reported in this paper.

## Data Availability

Data will be made available on request.
